# Outcomes of Urgent Suspected Cancer, Urgent, and Routine Skin Lesion Referrals Assessed via Teledermoscopy Service: A Retrospective Analysis

**DOI:** 10.7759/cureus.95721

**Published:** 2025-10-29

**Authors:** Sirag Elaribi, Ausama Atwan

**Affiliations:** 1 Dermatology, Aneurin Bevan University Health Board, Newport, GBR

**Keywords:** benign skin lesion, melanoma skin cancer, non-melanoma skin cancers, routine, teledermoscopy, urgent, urgent suspected cancer, wales

## Abstract

Background: Teledermoscopy facilitates the remote assessment of skin lesions by combining high-quality clinical and dermoscopic images. This technology enhances early detection of skin cancers by allowing specialists to evaluate lesions without the need for an in-person consultation. Beyond improving diagnostic accuracy, teledermoscopy supports efficient triage, prioritising patients who require urgent attention while reducing unnecessary clinic visits. Additionally, it enables longitudinal digital monitoring of lesions, allowing clinicians to track subtle changes over time, improve patient follow-up, and guide timely interventions.

Aim: To evaluate diagnostic outcomes and management decisions from primary care skin lesion referrals assessed via teledermoscopy across urgent suspected cancer (USC), urgent, and routine pathways.

Methods: A retrospective review was conducted of 1,203 patients referred to the South East Wales teledermoscopy service between September 2022 and December 2023. Referral letters and images were assessed, and cases were categorised by referral urgency. Final diagnoses and management outcomes were recorded, including discharge, minor operative procedures (MOPs), face-to-face (FTF) reviews, onward referrals, digital monitoring, or patient-initiated follow-up (PIFU).

Results: Nearly half of referrals were USC, with the remainder split between urgent and routine pathways. Seborrhoeic keratosis was the most frequent diagnosis, accounting for over a quarter of cases, while basal cell carcinoma was the most common malignancy identified. Probable melanomas and squamous cell carcinomas were concentrated in the USC pathway, but were also detected in urgent and routine referrals. Management outcomes varied: most routine cases were discharged, while a greater proportion of USC referrals required MOPs or FTF review. Digital monitoring was applied selectively and often led to safe discharge or escalation to FTF review. Histological confirmation of melanoma or SCC occurred in a small but essential proportion across all pathways.

Conclusion: Teledermoscopy safely reduced unnecessary FTF consultations and efficiently identified high-risk lesions. Most USC referrals were ultimately benign, highlighting the need to strengthen diagnostic confidence in primary care.

## Introduction

General practitioners (GPs) in England and Wales see more than 13 million patients each year for dermatological concerns [1]. In 2018 and 2019 alone, dermatology services recorded over 3.5 million outpatient and day-surgery attendances. Skin cancer now accounts for approximately half of all cancers diagnosed in England and Wales and continues to increase by about 8% annually. NHS dermatology units perform around 200,000 surgical procedures each year to remove malignant moles, lesions, and tumours.

According to NHS Digital data from the National Disease Registration Service (NDRS) Get Data Out programme, 224,000 skin cancers were diagnosed in England in 2019, with more than 1.4 million cases recorded between 2013 and 2019, making skin cancer the most common malignancy in the country [2]. This dataset, developed through collaboration between the British Association of Dermatologists (BAD) and the NDRS, represents the most up-to-date and comprehensive national record of skin cancer incidence. Between 2013 and 2019, reported cases rose from 177,677 to 224,092, marking a 26.1% increase over six years. This rise likely reflects a combination of factors, including an ageing population, increased sun exposure, and improved cancer detection and registration.

Teledermoscopy has emerged as a practical and effective strategy to address these mounting pressures. It allows dermatologists to remotely review high-quality clinical and dermoscopic images, facilitating accurate triage and timely identification of lesions requiring further investigation [3,4]. This approach enables more efficient use of specialist resources by distinguishing benign from suspicious lesions early, thereby reducing unnecessary face-to-face consultations. When integrated with digital monitoring, teledermoscopy also allows clinicians to track lesion changes over time and intervene promptly if malignant transformation is suspected.

Evidence indicates that teledermoscopy is a reliable and cost-, time-, and resource-saving method for assessing two-week-wait referrals and other high-risk skin cancer pathways [3,5]. A systematic review of seven studies involving 1,588 lesions (638 malignancies) reported that teledermatology using photographic images achieved a pooled sensitivity of 94.9% and specificity of 84.3% for detecting any skin cancer, comparable to in-person dermatologist assessment. Another systematic review of 21 studies found that teledermatology was consistently associated with reduced waiting times, earlier assessments, and high patient satisfaction, with many patients expressing a willingness to pay for access to such services.

## Materials and methods

Study design

This retrospective study assessed the effectiveness of teledermoscopy in managing skin lesion referrals. A total of 1,203 patients referred to the teledermoscopy service in South East Wales between September 2022 and December 2023 were included in the analysis. The inclusion criteria were referrals from primary care that provided a detailed clinical history and description of the lesion, accompanied by both clinical and dermoscopic images, and lesions that had not been previously assessed by a consultant dermatologist. The exclusion criteria were referrals lacking a comprehensive history or missing clinical and/or dermoscopic photos, as well as cases where another consultant had already reviewed the lesion. Referral letters, along with the accompanying clinical and dermoscopic images, were systematically reviewed by a consultant dermatologist with specialist expertise in dermoscopy and skin cancers. Features suggestive of malignancy - including an atypical pigment network, blue-white veil, polymorphous vessels, ulceration, arborising blood vessels, and keratinised or crusted lesions - were carefully evaluated to differentiate benign from malignant lesions. This methodology was designed to evaluate the diagnostic accuracy and management outcomes associated with teledermoscopic assessment. The likely diagnosis and recommended management plan were documented on an Excel spreadsheet (Microsoft, Redmond, WA, USA), along with the patients’ initials, hospital number, and the urgency of referral as indicated by the primary care referrer.

Data collection and analysis

The data for this study were collected from a teledermoscopy reporting spreadsheet provided by a consultant dermatologist with a special interest in teledermoscopy and skin cancers. The spreadsheet served as a repository of detailed information on referrals, diagnoses, and subsequent management plans of the 1,203 patients.

Each referral was categorised into one of three urgency groups: urgent suspected cancer (USC), urgent, or routine. This classification is based on triage decisions made by primary care providers.

The clinical and dermoscopic images submitted for each patient were reviewed, and diagnoses were recorded into specific categories. These included seborrhoeic keratosis (SK), basal cell carcinoma (BCC), benign naevi, actinic keratosis (AK), Bowen disease, melanoma, and squamous cell carcinoma (SCC). By organising diagnoses within each urgency category, the study was able to identify patterns in the distribution of benign and malignant conditions across the different referral pathways.

Following the diagnostic review, patients were assigned individualised management plans based primarily on the presence or absence of skin cancer features identified in both the clinical and dermoscopic images. Additional factors, including the patient age, past medical history of skin cancer, lesion progression, reported symptoms such as tenderness or bleeding, significant sun exposure or sunbed use, and immunosuppression status, were also taken into account to guide appropriate management decisions. These ranged from direct discharge to more intensive actions such as booking for minor operative procedures (MOPs), scheduling face-to-face (FTF) consultations, or referring to other surgical specialties (e.g., plastic, maxillofacial, or orthopaedic surgery). Digital monitoring was employed in cases requiring ongoing observation, and one patient was recommended for patient-initiated follow-up (PIFU). These decisions were linked back to the urgency category and clinical diagnosis, providing a comprehensive view.

Additionally, the outcomes of repeated photography, or digital monitoring, were assessed, and the diversion rates from outpatient consultations were calculated for all referral groups.

## Results

Pattern of teledermoscopy referrals

**Figure 1:**
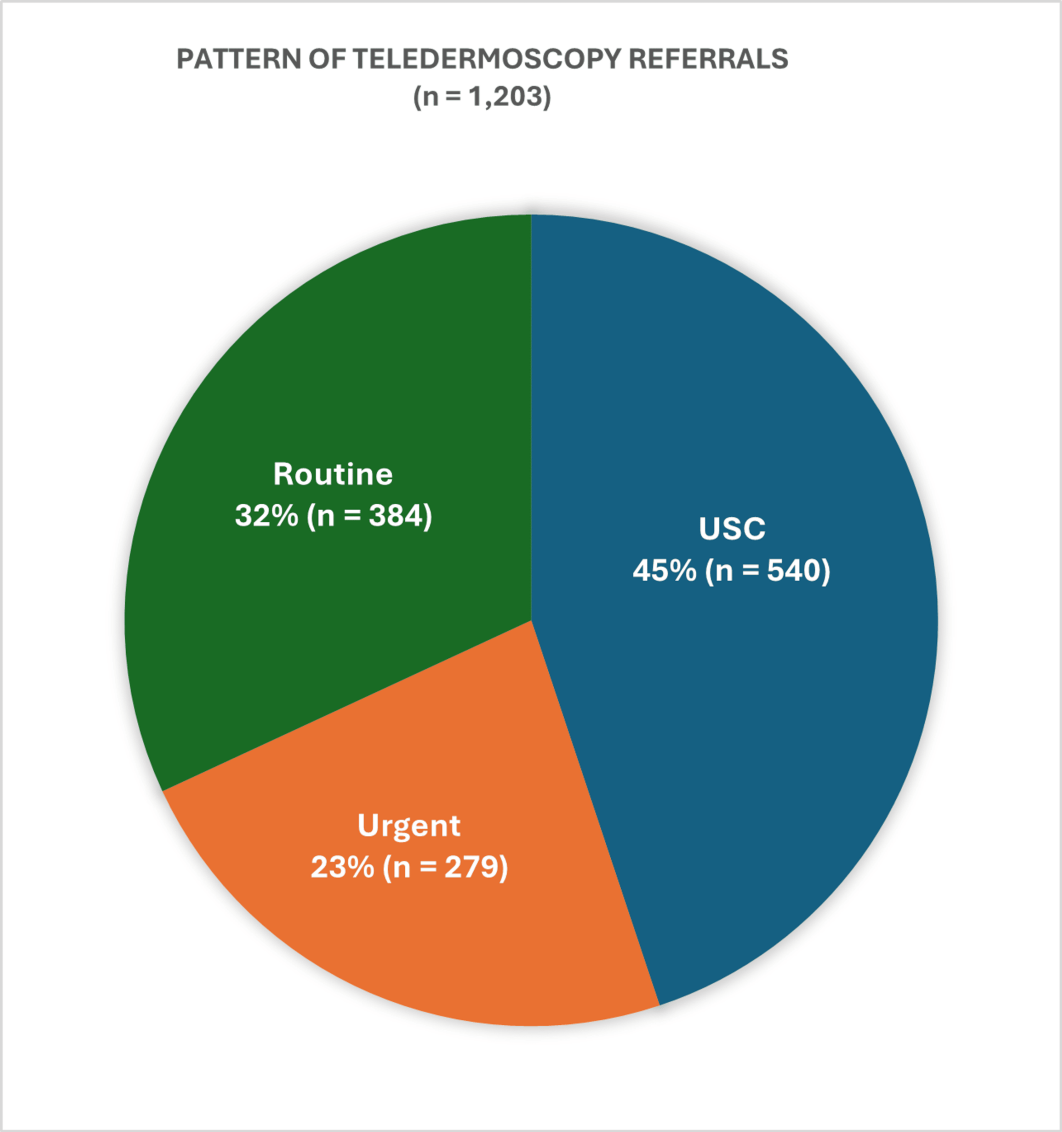
Pattern of Teledermoscopy Referrals USC: urgent suspected cancer

Among the 1,203 referrals reviewed in this study, the categorisation of urgency revealed notable distinctions in the patient population (Figure 1). Of these, 44.9% (n = 540) of the referrals were classified as USC. Meanwhile, 23.1% (n = 279) were categorised as urgent referrals, and 32% (n = 384) of the referrals fell into the routine category.

Diagnoses across the teledermoscopy referrals

The evaluation of diagnoses across the various referral categories revealed that SK was the most frequently identified condition, accounting for a significant portion of the cases: 25.9% (n = 140) in USC referrals, 27.6% (n = 77) in urgent referrals, and 25.8% (n = 99) in routine referrals. This finding highlights the high prevalence of benign skin lesions within the referral population. Following SK, BCC ranked as the second most frequently diagnosed condition among urgent and routine groups, representing 18.6% (n = 52) and 19% (n = 73), respectively. Notably, BCC was observed in 15% (n = 81) of USC referrals, positioning it as the third most prevalent diagnosis after SK and combined cases of melanoma and SCC. These results underscore the importance of BCC as a common malignancy encountered in dermatological practice.

The prevalence of probable melanomas and SCCs varied significantly across the different urgency categories, with the highest incidence found in the USC group (Table 1). Specifically, 15.9% (n = 86) of referrals in this category were identified as probable cases of melanoma or SCC. In contrast, the urgent referral group exhibited a lower prevalence of 8.2% (n = 23), while the routine group had the least incidence at just 5% (n = 19). Benign melanocytic naevi were predominantly observed in the USC group, accounting for 14.4% (n = 78) of referrals in this category. In contrast, the incidence of benign naevi in both the urgent and routine referral groups was notably lower, each comprising approximately 9% (n = 35) of their respective populations.

**Table 1:** Diagnoses of All Referral Pathways Assessed via Teledermoscopy USC: urgent suspected cancer, SCC: squamous cell carcinoma

Diagnoses	USC (44.9%, n = 540)	Urgent (23.1%, n = 279)	Routine (32%, n = 384)
Seborrhoeic keratosis	25.9% (n = 140)	27.6% (n = 77)	25.8% (n = 99)
Basal Cell Carcinoma	15% (n = 81)	18.6% (n = 52)	19% (n = 73)
Benign Naevi	14.4% (n = 78)	9.3% (n = 26)	8.8% (n = 34)
Actinic keratosis	5.4% (n= 29)	7.2% (n = 20)	9% (n = 35)
Bowen Disease	2% (n = 11)	1.4% (n = 4)	1.8% (n = 7)
Melanoma / SCC	15.9% (n = 86)	8.2% (n = 23)	5% (n = 19)

Bowen disease and AK collectively represented a notable portion of the referrals across all categories, accounting for approximately 7% to 10% of the total cases examined.

Outcomes of teledermoscopy referral

**Figure 2:**
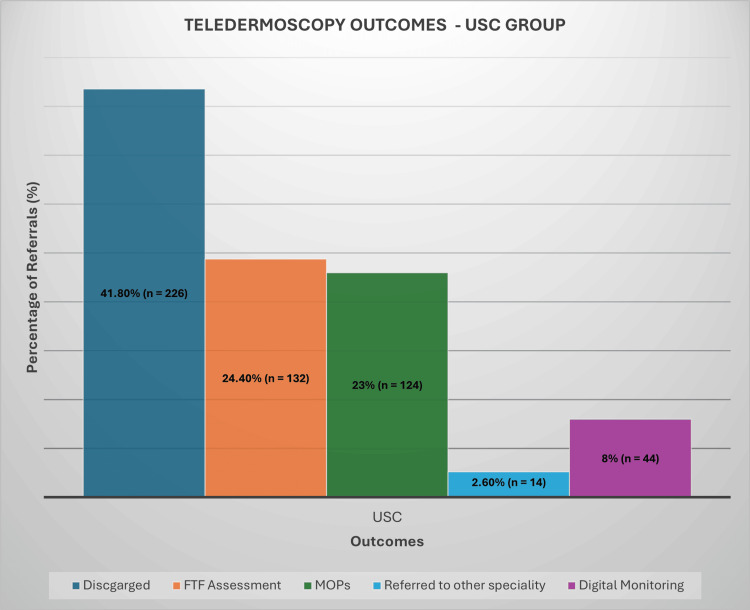
Teledermoscopy Outcomes - USC Group USC: urgent suspected cancer, FTF: face-to-face, MOPs: minor operative procedures

**Figure 3:**
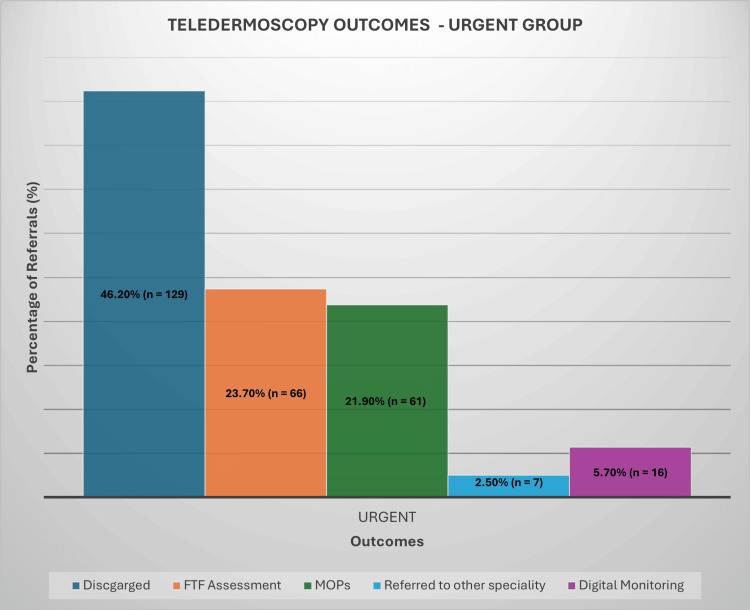
Teledermoscopy Outcomes - Urgent Group FTF: face-to-face, MOPs: minor operative procedures

**Figure 4:**
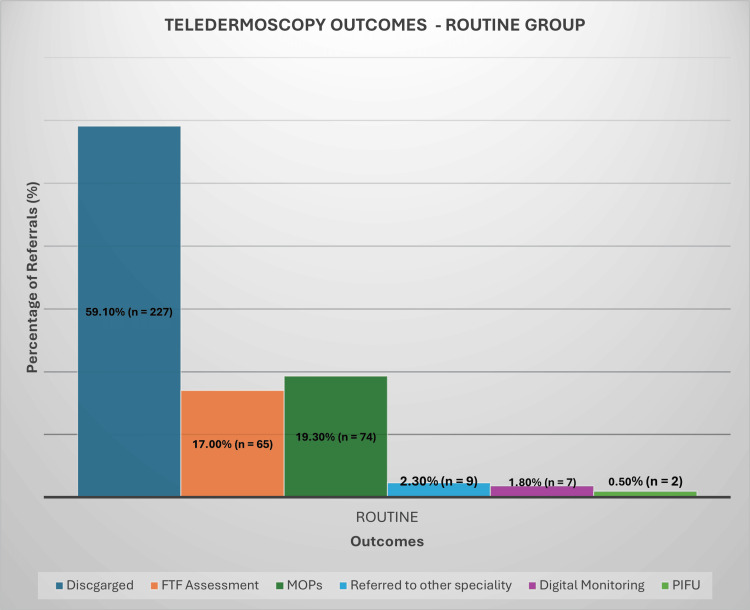
Teledermoscopy Outcomes - Routine Group FTF: face-to-face, MOPs: minor operative procedures

Management outcomes for the referrals varied significantly. In the USC group, a substantial proportion of patients (41.8%, n = 226) were discharged without requiring any further intervention (Figure 2). Additionally, 23% (n = 124) of the USC patients were scheduled for MOPs, while 24.4% (n = 132) required FTF consultations for further evaluation. In the urgent referral group, the outcomes were similarly revealing (Figure 3). A higher discharge rate of 46.2% (n = 129) indicated that many cases did not necessitate immediate action. Here, 21.9% (n = 61) of patients were booked for MOPs, while 23.7% (n = 66) were invited for FTF consultations. The routine referral category demonstrated the highest discharge rate at 59.1% (n = 227). Within this group, 19.3% (n = 74) were scheduled for MOPs, while 17% (n = 65) required further FTF evaluation (Figure 4).

Across all referral groups, a small percentage of cases, ranging from 2% to 3%, were forwarded to other surgical specialties, such as plastic, maxillofacial, or orthopaedic surgery. Digital monitoring was employed as a management strategy for selected cases, with 8% (n = 44) of USC referrals, 5.7% (n = 16) of urgent referrals, and 1.8% (n = 7) of routine cases utilising this approach. Notably, PIFU was recommended for only two patients in the routine referral group, representing less than 1% of cases.

Outcomes of digital monitoring (repeated photography)

**Figure 5:**
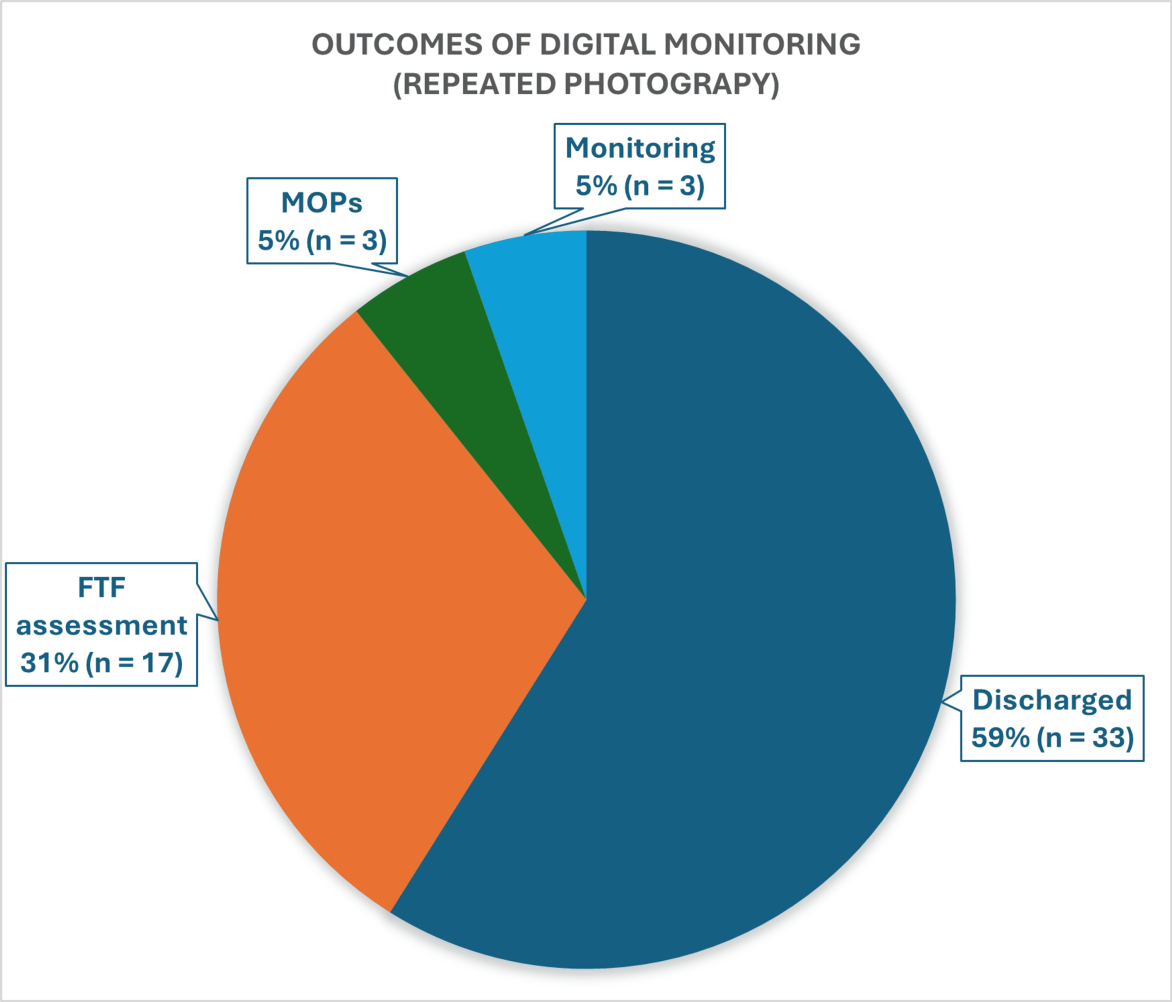
Outcomes of Digital Monitoring (Repeated Monitoring) FTF: face-to-face, MOPs: minor operative procedures

Among the patients who underwent digital monitoring (n = 56), a significant majority (59%, n = 33) were ultimately discharged (Figure 5). This high discharge rate indicates that many of these patients presented with benign lesions that did not warrant immediate intervention. Meanwhile, 31% (n = 17) of patients were invited for FTF assessments, and 5% (n = 3) of patients were scheduled for MOPs. Another 5% (n = 3) were recommended for ongoing digital monitoring, indicating a cautious approach for cases that required continued observation due to potential changes in the lesions over time.

Notably, among those patients undergoing repeated photography for digital monitoring, benign melanocytic naevi emerged as the most common diagnosis (35.7%, n = 20). This finding highlights the effectiveness of digital monitoring in managing these benign conditions.

Biopsy-proven melanoma and SCCs among the teledermoscopy referrals

Biopsy-proven melanomas and SCCs were identified in 1.3% (n = 5/384) of routine referrals, 1.8% (n = 5/279) of urgent referrals, and 5.4% (n = 29/540) of USC referrals. These findings highlight a significantly higher prevalence of biopsy-confirmed malignancies within the USC group, emphasizing the crucial role of this referral pathway in detecting high-risk lesions that require prompt evaluation and management.

Outpatient diversion rate for the teledermoscopy referrals

**Figure 6:**
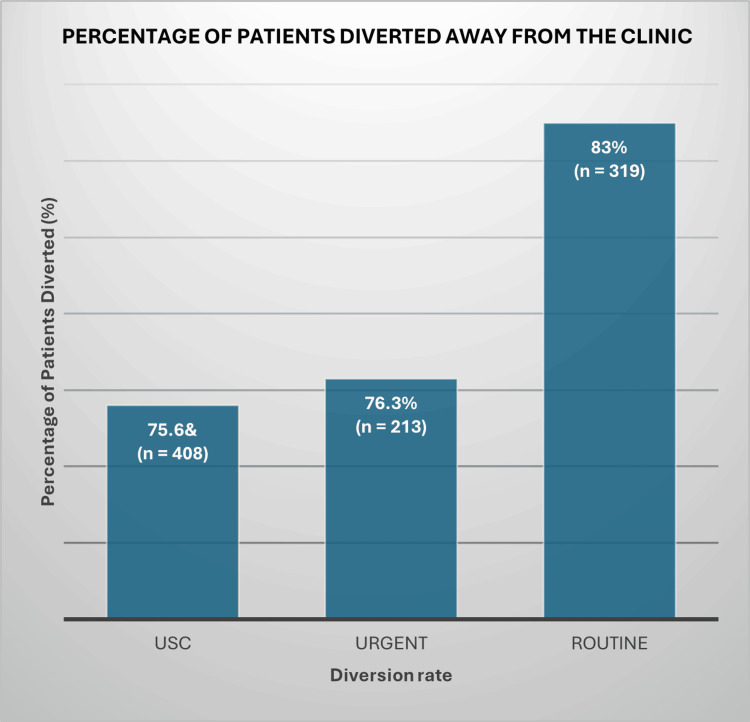
Outpatient Diversion Rate for the Teledermoscopy Referrals USC: urgent suspected cancer

The outpatient diversion rate, which indicates the percentage of referrals managed without the necessity for FTF consultations, was highest in the routine referral group at 83% (n = 319) (Figure 6). This was closely followed by the urgent referral group, which achieved a diversion rate of 76.3% (n = 213), while the USC group recorded a rate of 75.6% (n = 408). These results highlight the effective management of a significant number of patients remotely, particularly among those classified as routine cases. 

## Discussion

Teledermoscopy has become an integral component of modern dermatology practice, enabling rapid specialist assessment and improving the efficiency of referral pathways [6]. It allows many patients with suspected skin cancer to be safely managed without the need for in-person consultation [7,8]. The service has also proved invaluable during periods of restricted clinical access, such as the COVID-19 pandemic, by maintaining continuity of care and reducing diagnostic delays [9].

An evaluation of an NHS-compliant teledermatology service implemented across 12 GP practices during the COVID-19 pandemic demonstrated substantial efficiency gains and high patient satisfaction. Between January 2020 and May 2021, 88% of referrals made through the teledermatology platform avoided FTF appointments, reducing waiting times for lesion review from 10-47 days to less than one day. Most patients (92%) described the service as quick and efficient, valuing its convenience and speed, although a minority still preferred in-person consultations [10].

Findings from our study further highlight the value of teledermoscopy in the assessment and management of skin lesions. The predominance of SK as the most common diagnosis across all referral pathways reflects the high prevalence of benign skin conditions in clinical practice. This underscores the importance of enhancing diagnostic confidence among primary care providers to help them better recognise benign lesions, reduce patient anxiety, and minimise unnecessary referrals to secondary care.

A considerable proportion of USC referrals were for non-melanoma and non-SCC lesions, suggesting an over-reliance on the USC pathway. This is likely attributable to limited dermatology and dermoscopy training among GPs, combined with the rising incidence of skin cancer in the UK, which has encouraged a cautious approach to referrals. Given the medico-legal implications of missing a melanoma or SCC, many clinicians prefer to refer any lesion with suspicious features, even when the likelihood of malignancy is low. Conversely, a small but important proportion (5%, n = 19) of routine referrals involved lesions later confirmed as melanoma or SCC, indicating that improved dermatology and dermoscopy training in primary care could enhance triage accuracy and ensure timely identification of malignant cases.

The consistently high outpatient diversion rates across all referral categories demonstrate the effectiveness of teledermoscopy in alleviating pressure on dermatology outpatient services. This approach enhances clinic efficiency and spares patients unnecessary face-to-face consultations. Moreover, digital monitoring enables clinicians to identify subtle changes in lesions over time that may signal malignant transformation - such as the development of an irregular pigment network, atypical globules, arborising vessels, or ulceration - thereby facilitating early intervention and improving patient outcomes. Notably, 75.6% (n = 408) of USC referrals were managed entirely without FTF assessment, highlighting the reliability of high-quality clinical and dermoscopic imaging in supporting safe, remote decision-making. The higher diversion rates observed in urgent (76.3%, n = 213) and routine (83%, n = 319) referrals likely reflect the predominance of benign or easily managed conditions, for which teledermoscopy provides sufficient diagnostic confidence to guide management remotely. Digital monitoring also proved valuable for tracking lesion evolution, particularly in melanocytic and borderline cases, supporting the long-term safety and practicality of teledermoscopic follow-up.

Evidence from large-scale studies aligns with these findings. A retrospective cross-sectional study from São Paulo, Brazil, involving 30,976 individuals and 55,624 lesions, reported that 53% of cases were managed effectively within primary care via teledermatology, 43% required in-person review, and 4% were referred for biopsy [11]. The introduction of teledermatology led to a 78% reduction in waiting times for FTF appointments. Similarly, another study analysing 571 dermatology referrals found that 72% of patients were managed successfully without in-person consultation, while 28% required FTF review [12]. Consistent with these results, Hamid et al. evaluated 383 teledermatology referrals for suspected skin malignancy and found that 83.7% of cases were managed entirely remotely [13].

Finally, the detection of biopsy-proven melanoma and SCC in only 1.3% (n = 5) of routine, 1.8% (n = 5) of urgent, and 5.4% (n = 29) of USC referrals underscores the relatively low prevalence of malignant lesions outside the USC pathway. While benign conditions predominate, these findings emphasise the continuing need for enhanced diagnostic training and support in primary care to ensure appropriate triage and reduce unnecessary escalation through USC pathways. Targeted educational initiatives, such as hands-on dermoscopy workshops, British Association of Dermatologists (BAD)-accredited skin lesion recognition courses, and online modules in dermatological diagnosis, can significantly improve diagnostic confidence and reduce inappropriate referrals by helping clinicians recognise key dermoscopic features, including pigment networks, globules, vascular patterns, and ulceration. However, implementing such programmes remains challenging due to time constraints in primary care, limited funding for professional development, and inadequate access to dermatoscopes and IT infrastructure to support the integration of teledermoscopy into routine practice.

## Conclusions

This study demonstrates the effectiveness of teledermoscopy in managing skin lesion referrals from primary care. The predominance of benign diagnoses, particularly seborrhoeic keratosis, across all referral pathways underscores the potential to improve diagnostic accuracy and confidence among primary care clinicians. High outpatient diversion rates further highlight the value of teledermoscopy in enabling safe, efficient remote management while substantially reducing the need for face-to-face consultations.

To build on these findings, future research should explore the long-term impact of teledermoscopy on patient outcomes, its cost-effectiveness across different healthcare systems, and its role in sustaining diagnostic accuracy over time. Strengthening diagnostic capabilities in primary care through targeted initiatives - such as hands-on dermoscopy workshops, BAD-accredited skin lesion recognition courses, and teledermoscopy-based training modules - could further optimise referral pathways, enhance early cancer detection, and improve overall resource utilisation within dermatology services.
